# Human *O*-GlcNAcase Uses a Preactivated Boat-skew Substrate Conformation for Catalysis. Evidence from X-ray Crystallography and QM/MM Metadynamics

**DOI:** 10.1021/acscatal.3c02378

**Published:** 2023-10-10

**Authors:** Martín Calvelo, Alexandra Males, Matthew G. Alteen, Lianne I. Willems, David J. Vocadlo, Gideon J. Davies, Carme Rovira

**Affiliations:** † Departament de Química Inorgànica i Orgànica & IQTCUB, Universitat de Barcelona, Martí i Franquès 1, 08028 Barcelona, Spain; ‡ York Structural Biology Laboratory, Department of Chemistry, The University of York, Heslington, York YO10 5DD, United Kingdom; § Department of Chemistry & Department of Molecular Biology and Biochemistry, Simon Fraser University, Burnaby, British Columbia V5A 1S6, Canada; ∥ Institució Catalana de Recerca i Estudis Avançats (ICREA), Passeig Lluís Companys, 23, 08020 Barcelona, Spain

**Keywords:** *O*-glycans, enzyme catalysis, catalytic reaction mechanism, glycoside hydrolases, hexosaminidases, quantum mechanics/molecular mechanics, metadynamics

## Abstract

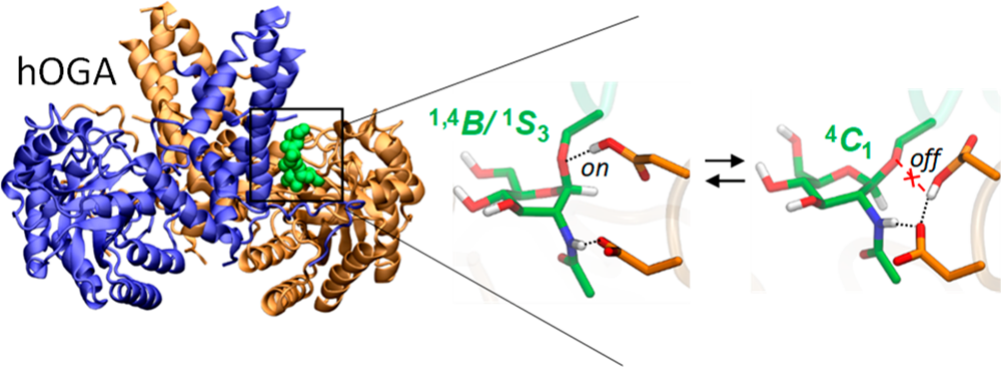

Human *O*-linked β-*N*-acetylglucosaminidase (hOGA) is one of the two enzymes involved in nuclear and cytoplasmic protein O-GlcNAcylation, an essential post-translational modification. The enzyme catalyzes the hydrolysis of the GlcNAc-*O*-(Ser/Thr) glycosidic bonds via anchimeric assistance through the 2-acetamido group of the GlcNAc sugar. However, the conformational itinerary of the GlcNAc ring during catalysis remains unclear. Here we report the crystal structure of wild type hOGA in complex with a nonhydrolyzable glycopeptide substrate and elucidate the full enzyme catalytic mechanism using QM/MM metadynamics. We show that the enzyme can bind the substrate in either a chair- or a boat-like conformation, but only the latter is catalytically competent, leading to the reaction products via ^1,4^*B*/^1^*S*_3_ → [^4^*E*]^‡^ → ^4^*C*_1_ and ^4^*C*_1_ → [^4^*E*]^‡^ → ^1,4^*B*/^1^*S*_3_ conformational itineraries for the first and second catalytic reaction steps, respectively. Our results reconcile previous experimental observations for human and bacterial OGA and will aid the development of more effective OGA inhibitors for diseases associated with impaired *O*-GlcNAcylation.

*O*-GlcNAcylation, the attachment of a β-*N*-acetylglucosamine (GlcNAc) sugar to serine or threonine residues in proteins of higher eukaryotes, is a dynamic reversible process that is regulated within cells.^1^ In mammals, *O*-GlcNAcylation and its hydrolytic removal are catalyzed by two opposing enzymes, *O*-GlcNAc transferase (OGT) and *O*-GlcNAc hydrolase (OGA).^2^ The former attaches GlcNAc to the hydroxyl group of Ser or Thr residues in proteins, with inversion of configuration, whereas the latter removes it, with retention of configuration.^3^ Decreased *O*-GlcNAcylation has been associated with diseases including cancer,^4^ obesity,^5^ and neurodegeneration, with OGA being a therapeutic target for Alzheimer’s disease.^6−8^ Therefore, there is enormous interest in understanding the molecular and chemical basis of *O*-GlcNAcylation.

OGA is a multidomain enzyme comprising a nonfunctional acetyltransferase domain, a glycoside hydrolase domain that belongs to CAZy family 84 (GH84), and a structurally important stalk domain involved in dimerization.^9^ A variety of crystal structures in complex with substrate analogues and inhibitors have been reported. These structures include bacterial orthologues of OGA (mainly *Bacteroides thetaiotaomicron* and *Clostridium perfringens*) in complex with a slow substrate analogue (3,4-difluorophenyl 2-deoxy-2-difluoroacetamido-β-glucoside) and several inhibitors, including 5F-oxazoline, PUGNAc, and its derivatives.^10−12^ Structures of human OGA (hOGA) in complex with various inhibitors (Thiamet-G, PUGNAc–imidazole, and pyrrolidine derivatives)^11,13^ as well as hOGA variants in complex with synthetic glycopeptides have also been reported.^13,14^

GH84 enzymes operate via anchimeric assistance by the 2-acetamido group of the GlcNAc sugar of the substrate (Scheme 1).^15−20^ The reaction mechanism involves two distinct chemical steps (named “cyclization” and “ring opening”, respectively), leading to hydrolysis with retention of configuration at the anomeric center. In the first step, a carboxylate residue (D174 in hOGA) polarizes and orients the substrate NHAc group, while the NHAc oxygen atom attacks the anomeric carbon to form a cyclic oxazoline/oxazolinium ion intermediate. Simultaneously, an acidic residue (D175 in hOGA) acts as a general acid/base, assisting leaving group departure through the transfer of a proton to the leaving group oxygen. In the second step, D175, which now acts as a general base, activates a water molecule to attack the anomeric carbon to release the products GlcNAc and the peptide.

**Scheme 1:**
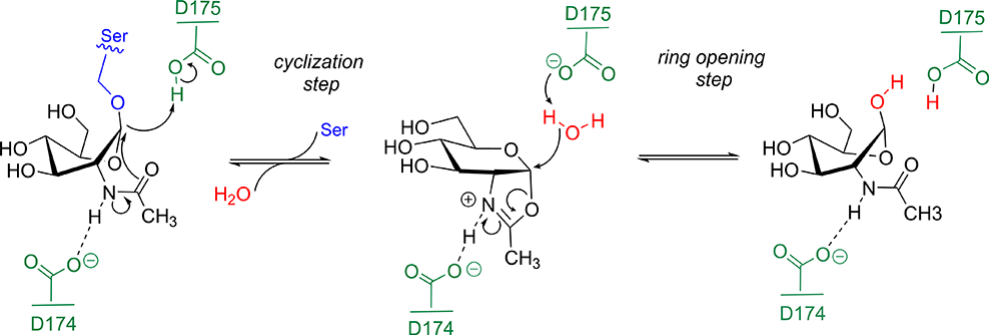
Neighboring Group Participation Mechanism Catalyzed by Family GH84 Glycoside Hydrolases (hOGA Residue Numbering)

A precise delineation of the hOGA mechanism would be useful to boost the rational design of enzyme inhibitors to control *O*-GlcNAcylation. However, the conformations of the GlcNAc substrate throughout the entire reaction, from the Michaelis complex to the products of the reaction, remain unclear. A previous study by some of us on bacterial OGAs showed that mutation of the acid/base residue can convert the enzyme from hydrolase to phosphorylase.^19^ The first step of the reaction mechanism, of relevance in this work, was not investigated. Concerning hOGA, a recent structural study by Li et al. using the D175N variant in complex with a synthetic glycopeptide revealed that the GlcNAc ring is in a type of half-chair conformation (^4^*H*_5_) in one monomer and an envelope conformation (^4^*E*) in the other monomer.^14^ However, other recent structures of hOGA with a series of inhibitors reported a quite distinct boat-type conformation (^1,4^*B*).^11^ Inhibitor complexes of bacterial OGA, including a complex with a slow substrate analogue,^10^ also reported a ^1,4^*B*/^1^*S*_3_ conformation. Further complicating the situation, a recent computational study of hOGA proposed an unusual, inverted chair conformation (^1^*C*_4_),^20^ which has not yet been observed experimentally for any β-*N*-acetylglucosaminidase. Therefore, there is no consensus regarding the conformation adopted by the substrate at the Michaelis complex of hOGA and its full conformational itinerary during catalysis.

Here we report a crystal structure of wild type hOGA in complex with a nonhydrolyzable glycopeptide substrate in which the peptide is *S*-GlcNAcylated at a cysteine residue. We use this structure to reconstruct the *O*-GlcNAcylated peptide and determine the conformational free energy landscape (FEL) of the reactive GlcNAc, as well as the entire catalytic mechanism at atomic detail, by means of QM/MM metadynamics simulations.

Crystals of WT hOGA, protein produced by coexpression of the glycoside hydrolase domain (residues 11–396) and the stalk domain (535–715),^11^ were seeded with a known peptide substrate^21^ derived from casein kinase II (CKII) (339-YPGGSTPV**S**SANMM-352) and the corresponding CKII-Cys-GlcNAc glycopeptide. This peptide was produced by *S-*GlcNAcylation using OGT (see the Supporting Information).^22^ Crystals of WT hOGA and the CKII-Cys-GlcNAc complex diffracted to a resolution of 2.5 Å (Table S1). The resulting hOGA-CKII-Cys-GlcNAc structure (Figure 1A) shows density at the active site, corresponding to GlcNAc-Cys. GlcNAc was bound in a ^1,4^*B*/^1^*S*_3_ conformation,^23^ with several interactions between the GlcNAc moiety and hOGA residues, from N2, O7, O3, O4, and O6 of GlcNAc, to D174, N280, K98, G67, N313, and D285 that have been previously characterized (Figure S2).^11^ The peptide could be partially built into the active site of one monomer. Surprisingly, only two of the 14 residues of the peptide could be modeled, Cys347 and Ser348, and no interactions were observed between the CKII peptide and the active site residues of hOGA. Such weak interactions between the CKII peptide and hOGA could allow for flexibility and accommodation of the vast range of known protein targets within the active site. Similar disorder of the peptide was observed in the structures of hOGA in complex with TAB1, ELK1, α-Crystallin B and Lamin B,^14^ where very few amino acids for these peptides could be modeled. The CKII peptide binds in the same orientation to p53-Ser-GlcNAc observed in complex with the catalytically incompetent D175N hOGA (PDB 5UN8)^13^ (Figure S2). The direction of peptide binding is “opposite” to the published OGA structures with alpha-crystalline B and ELK1.^14^

**Figure 1:**
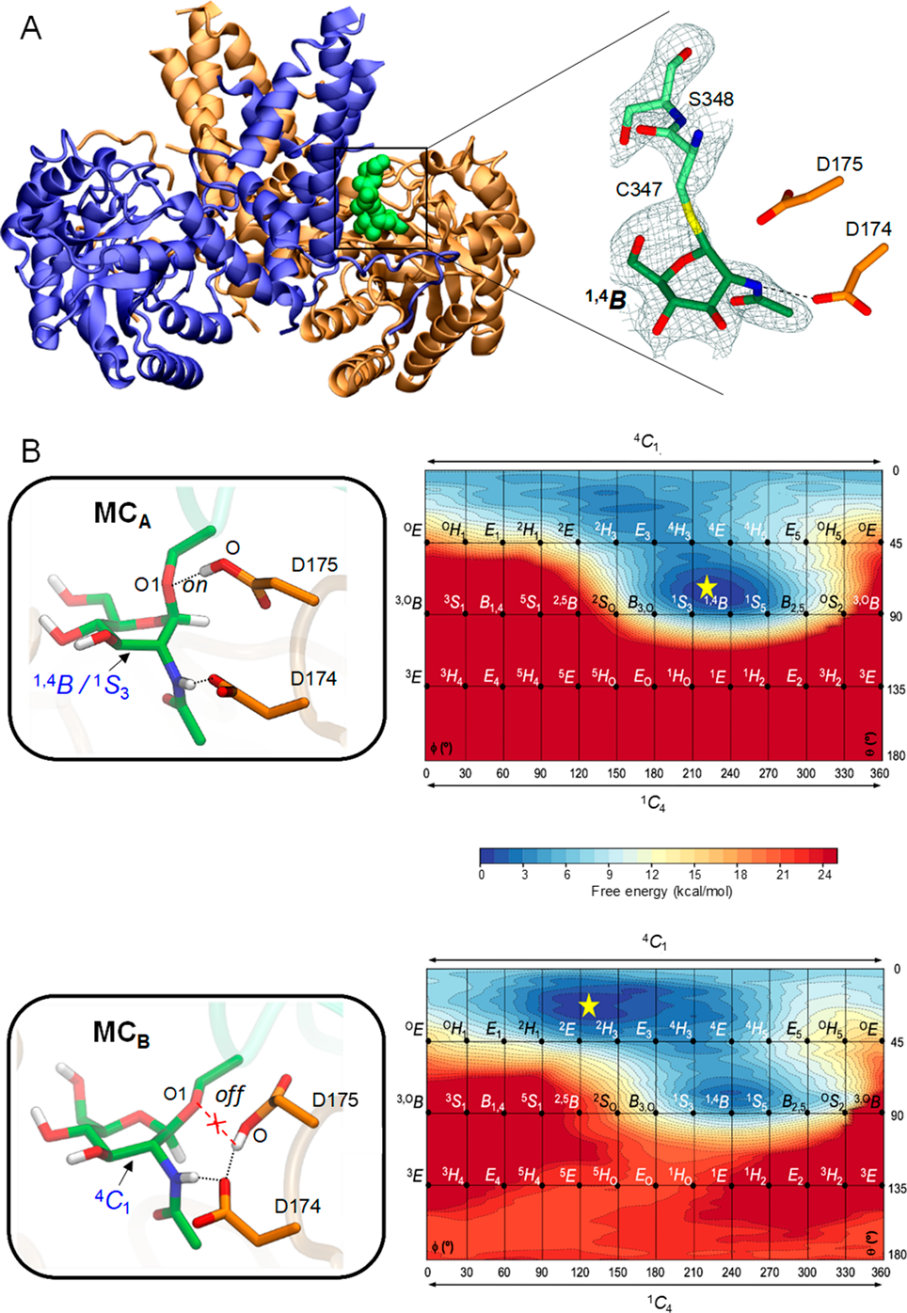
(A) Crystal structure of WT hOGA in complex with CKII-Cys-GlcNAc bound in one active site of the homodimer (monomers in purple, blue, and gold). The maximum-likelihood/σA-weighted 2*F*_o_–*F*_c_ map, shown in gray, is clipped to GlcNAc and the peptide separately; it is contoured at 0.13 and 0.10 e Å^–3^, respectively. (B) Active site structure in the two alternative configurations of the Michaelis complex, MC_A_ and MC_B_, along with the corresponding conformational free energy landscape (FEL, in Mercator representation) of the GlcNAc. Hydrogen atoms attached to carbon atoms have bene omitted for clarity, except for C1–H1. The conformational FELs were obtained from QM/MM metadynamics simulations using Cremer–Pople puckering coordinates as collective variables. Isolines are 1 kcal/mol.

The structure of hOGA in complex with CKII-Cys-GlcNAc, in which all atoms of the GlcNAc residue are well ordered (average *B* factor of 39 Å^2^), was used to start molecular dynamics (MD) simulations, using AMBER^24^ (Supporting Information). The S atom of Cys was replaced by O, and the terminal chain of the solvent-exposed part of the glycopeptide was reconstructed from the structure of D175N hOGA in complex with p53-Ser-GlcNAc (PDB 5UN8). The two protein subunits were taken into account, as required for enzyme activity.^13^ The system was relaxed and equilibrated by extensive MD simulations (1 μs), during which the substrate remained in place, with the assisting D174 residue forming a persistent hydrogen bond with the NH group of the acetamido substituent of GlcNAc (Figure S4). Interestingly, the acid/base residue D175 was found to exhibit two conformations, either interacting with the glycosidic oxygen (Michaelis Complex A, MC_A_, see Figure 1B) or interacting with the assisting residue (Michaelis Complex B, MC_B_), in which case the carboxylic acid group of D175 adopts the less favorable anti conformation.^25^ The transformation of MC_B_ into MC_A_ brings the proton of D175 close to the glycosidic oxygen (1.9 Å), enabling general acid catalysis and departure of the leaving group and promoting the cyclization of the GlcNAc. These two conformations were not obtained in MD simulations using CKII-GlcNAc-Cys as a substrate. In this case, D175 was oriented toward the S atom, resulting in conformations highly similar to those observed in the crystal structure (Figure S10).

To investigate the active site dynamics associated with the interconversion between MC_A_ and MC_B_, we turned to DFT-based MD, since classical force fields often fail to reproduce conformations of pyranose rings.^26,27^ The QM/MM metadynamics approach^28,29^ was used to bring the system from MC_B_, in which the two catalytic residues interact, to MC_A_, in which D175 points productively toward the glycoside oxygen (Figure S11A). In addition, we computed the conformational FEL of the GlcNAc sugar at the two end points, MC_A_ and MC_B_, with respect to Cremer–Pople puckering coordinates.^30^ The simulations were performed with the CP2K program,^31^ along with the PBE exchange-correlation functional,^32^ as in previous work on GHs.^26,33,34^ The QM region was taken as the GlcNAc-Ser moiety of the glycopeptide and the two catalytic residues (D174 and D175). An active-site lysine residue (K98) that forms a salt-bridge interaction with the assistant residue (D174) and could significantly influence its p*K*_a_ was also included (55 QM atoms, 187862 MM atoms, Figure S5A).

The simulations show that states MC_A_ and MC_B_ are isoenergetic, separated with a relatively low energy barrier (<12 kcal/mol; Figure S11). Interestingly, the conformational FEL of GlcNAc at both states (Figure 1B) reveals that, whereas the sugar at MC_B_ adopts a ^4^*C*_1_ conformation, it shifts to a conformation that is intermediate between ^1,4^*B* and ^1^*S*_3_ at MC_A_, in which the leaving group is in an axial orientation, thus being preactivated for catalysis. The latter is in agreement with previous structures of bacterial OGA in complex with inhibitors and a slow substrate analogue.^10,11^ It is also in agreement with the present experimental structure of wild-type hOGA with the CKII-Cys-GlcNAc glycopeptide, in which the two catalytic residues are in a configuration similar to that of MC_A_ (Figure 1A). The ^4^*H*_5_ and ^4^*E* conformations observed in the structure of D175N hOGA^14^ are higher in energy by 4–5 kcal/mol at both MC_A_ and MC_B_. Therefore, our simulations reveal that, while the assisting residue is entirely fixed in place and forms an interaction with the NHAc group, the acid/base residue is quite mobile and can adopt two possible orientations (on/off). Such motion of the acid/base residue not only helps to orient it properly for catalysis but also modulates the conformational landscape of the GlcNAc sugar, which changes from a ^4^*C*_1_ chair to a preactivated ^1,4^*B*/^1^*S*_3_ conformation as the acid/base starts interacting with the glycosidic oxygen.

The enzyme configuration in the preactivated MC_A_ state was used to model the first step of the enzymatic reaction (Scheme 1). We considered one collective variable describing both the glycosidic bond cleavage and protonation of the glycosidic bond assisted by the acid–base residue (D175), as well as the attack of the oxygen of the O_NHAc_ to the sugar anomeric carbon (CV = (*d*_(OH)–_D175__ – *d*_O1···H_D175__) + (*d*_C1–O1_ – *d*_C1···ONHAc_)]. The QM/MM metadynamics simulation successfully drove the system from the MC_A_ state to the reaction intermediate, with a reaction free energy barrier of 16.1 kcal/mol (Figure 2A). This value is in agreement with the one that can be estimated from experimental reaction rates (16–18 kcal/mol).^15,35−37^ Our results show a better agreement with experiment compared to those obtained in the study by Xu et al., in which the simulations were initiated from an inverted chair conformation (^1^*C*_4_) of the GlcNAc moiety (data from reported coordinates and figures, but defined as ^4^*C*_1_ in the text).^20^ These disparities may arise from the differences in the pucker of the sugar ring, although the utilization of different levels of theory in the QM region (DFT in this work vs SS-DFTB in ref (20)) and method to find the reaction coordinate (dynamic QM/MM in this work vs static QM/MM in ref (20)) complicates the direct comparison.

**Figure 2:**
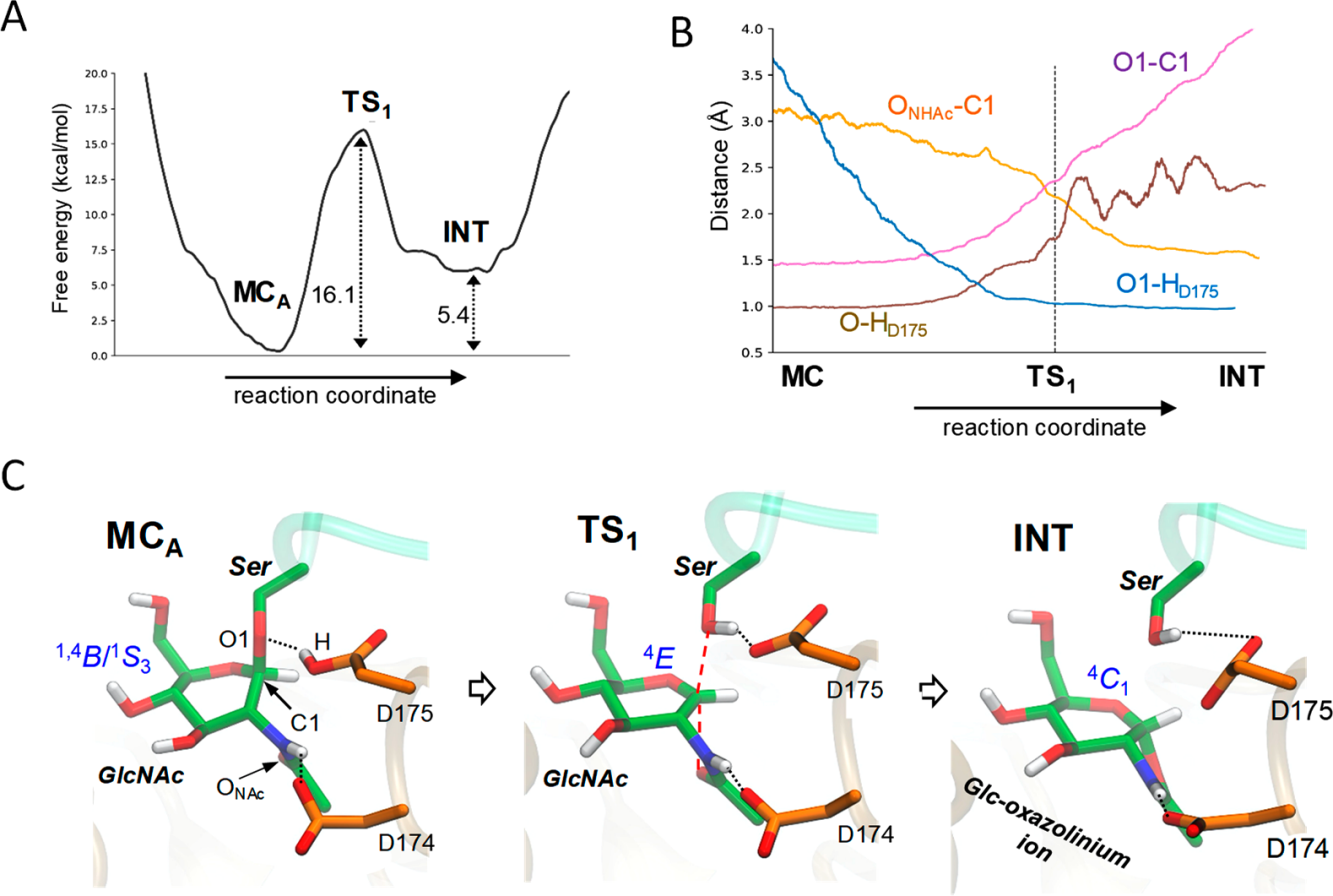
(A) Free energy profile. (B) Evolution of the main active site distances along the reaction coordinate. (C) Representative structures of the main states along the reaction coordinate for the first reaction step (cyclization). Hydrogen bonds are shown as dotted black lines, whereas dashed red lines indicate that covalent bonds are broken or formed.

Analysis of the structure of the enzyme along the reaction coordinate shows that the reaction starts with the lengthening of the glycosidic bond and concomitant proton transfer to the glycosidic oxygen from D175, followed by the approach of the NHAc oxygen to C1 (Figure 2B,C). This leads to the formation of an intermediate (INT), in which the NHAc hydrogen atom remains with the NHAc group; thus, it can be best described being an oxazolinium ion. An unbiased QM/MM simulation confirmed this result (Figure S12), as we also found in our previous work on an engineered bacterial OGA.^19^ These results are also consistent with the relative p*K*_a_ values of the aspartic acid side chain and the oxazolinium ion (3.9 and 7.7, respectively).^38^ Moreover, a persistent interaction between D174 and a lysine residue (K98) (Figure S6) likely decreases the p*K*_a_ of D174, which is again most consistent with the presence of an oxazolinium ion intermediate.^19^

Whereas the configuration of the sugar at MC_A_ is between ^1,4^*B* and ^1^*S*_3_, it changes to a ^4^*E* conformation at the TS, which displays the planar conformation around C1 that is typical of an oxocarbenium ion-like configuration.^39,40^ Consistent with its involvement in stabilization of the cationic TS, the endocyclic C1–O5 distance shrinks from 1.41 Å at MC_A_ to 1.31 Å at the TS, in which the C1–O bond is partially broken (2.3 Å) and the C1–O_NHAc_ bond is partially formed (2.2 Å; Figure 2B). The proton of the acid–base residue is much closer to the glycosidic oxygen (1.07 Å) than to the carboxylate group of D175 (1.53 Å); thus, protonation of the glycosidic bond is almost complete at the TS (Figure 2B,C). This observation is consistent with kinetic experiments showing a small negative β_lg_(V/K) value of −0.11, which suggests little negative charge on the glycosidic oxygen at the TS in the first step of the reaction.^41^ The pyranose then evolves toward a ^4^*C*_1_ conformation after the cyclization is complete. Therefore, the substrate follows a ^1,4^*B*/^1^*S*_3_ → [^4^*E*]^‡^ → ^4^*C*_1_ itinerary during the first reaction step.

To model the second step of the enzymatic reaction, hydrolysis of the oxazolinium ion intermediate, we removed the leaving peptide chain and relaxed the system using classical MD simulations, allowing water molecules to enter the active site. Analysis of the dynamics of water molecules visiting the active site (Figure 3A and Figure S13) shows that 75% of the time, there is a water molecule within 2 Å from both D175 and the anomeric carbon. We took a snapshot corresponding to this configuration to model the second step of the enzyme reaction by QM/MM metadynamics. We considered one CV that includes both the deprotonation of the water molecule by D175 and the nucleophilic attack of the water molecule (CV = (*d*_O_w_–H_w__ – *d*_O1_D175_···H_w_)_ + (*d*_C1–O_NHAc__ – *d*_(C1···O_w__)). The resulting free energy profile indicates a concerted reaction with a free energy barrier of 11.1 kcal/mol (Figure 3B). This value is lower than the one found for the first catalytic step, indicating that the formation of the reaction intermediate is rate-limiting, which is consistent with kinetic studies.^16^ As expected, the product state (P) is more stable than the reaction intermediate (6.8 kcal/mol), resulting in an exothermic process.

**Figure 3:**
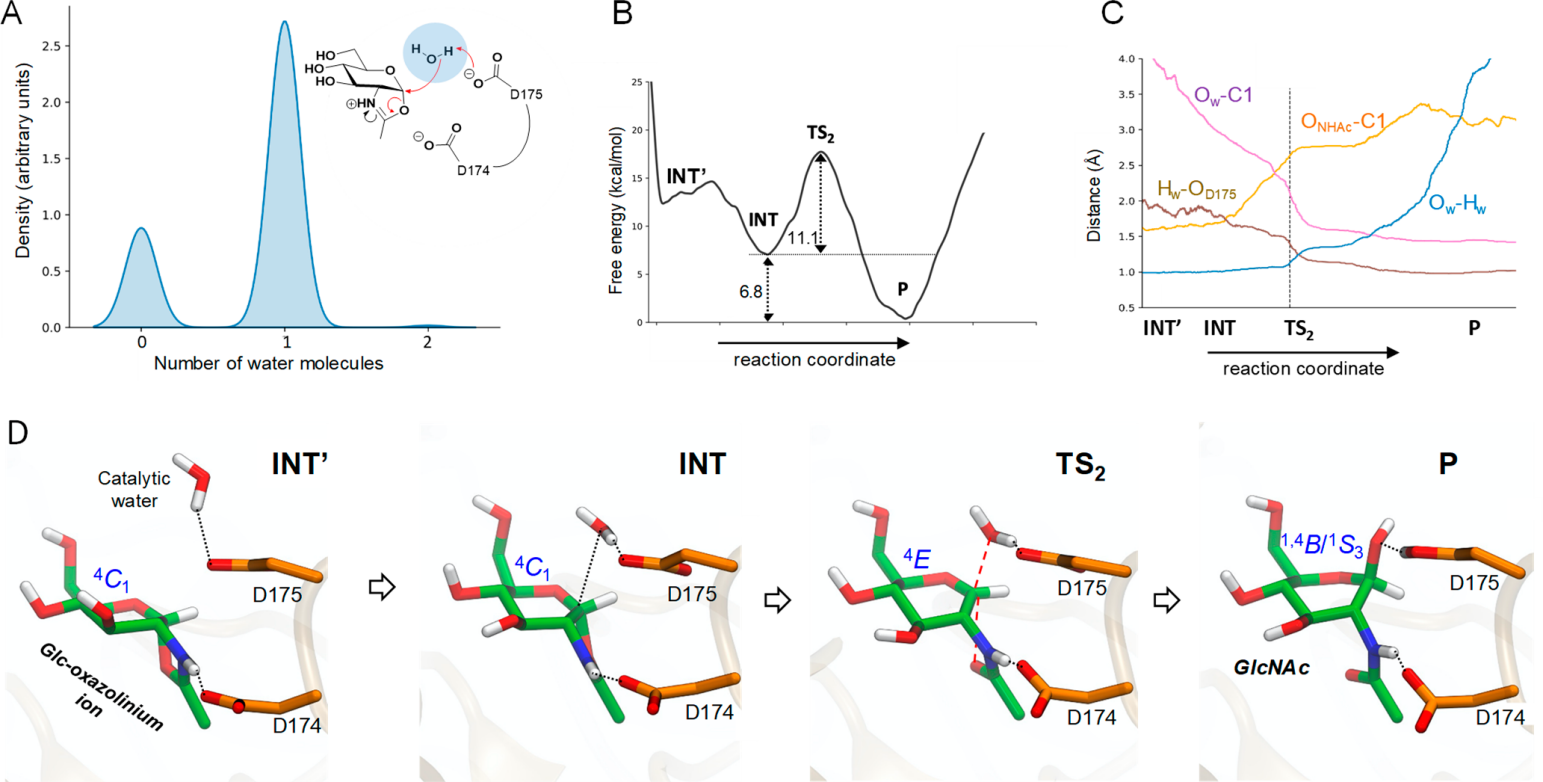
(A) Density distribution of the number of water molecules in a sphere of radius 2 Å centered at the geometrical center between the C1 atom of GlcNAc and the carboxylate group of D175. (B) Free energy profile. (C) Evolution of the main active site distances along the reaction coordinate. (D) Representative structures of the main states along the reaction coordinate for the second reaction step (ring opening).

Interestingly, the simulations captured an alternative state for the reaction intermediate, corresponding to the small minimum at the left-hand side of the free energy profile (INT′ in Figure 3B). In this state, the water molecule is not yet engaged with D175. Therefore, the evolution of the active site along the reaction coordinate (Figure 3D) shows that the reaction starts with the approach of the water molecule until it is bonded by D175. Subsequently, the C1–O_NHAc_ bond elongates and the water molecule starts to attack the anomeric carbon, reaching a TS state in which the sugar adopts a planar conformation (^4^*E*) and acquires oxocarbenium ion-type character. At the TS, the bond between the water molecule and the anomeric carbon is partially formed, that between the anomeric carbon and the O_NHAc_ atom is partially broken (2.14 and 2.56 Å, respectively, Figure 3C), and one water proton is being transferred to the acid–base residue (O_w_–H_w_ = 1.16 Å; H_w_–O_D175_ = 1.29 Å). Once the product state is reached, D175 is fully protonated. The conformation of the pyranose at P fluctuates between ^1,4^*B* and ^1^*S*_3_, as found in the productive MC. Therefore, the conformational itinerary of the second catalytic step can be described as ^4^*C*_1_ → [^4^*E*]^‡^ → ^1,4^*B*/^1^*S*_3_, which is the microscopic reverse of the itinerary for the first catalytic step.

In summary, the results of both X-ray crystallography and QM/MM simulations reported are consistent with hOGA using a preactivated ^1,4^*B*/^1^*S*_3_ conformation for catalysis. However, the substrate can easily undergo a conformational change to an unreactive chair conformation (^4^*C*_1_) in which the two catalytic residues are engaged in a hydrogen bonding interaction. Such conformational flexibility in the active site is probably the reason for the discrepant results obtained in previous structural studies regarding the substrate conformation. Our QM/MM simulations were also able to delineate the full catalytic mechanism from the Michaelis complex to the products of the enzymatic reaction, in which the GlcNAc is detached from the glycopeptide substrate. Gratifyingly, these findings that provide detailed structural insights into catalysis by hOGA are consistent and explain the results from enzyme kinetic studies. Our combined X-ray and simulation study shows that the two half-reactions involve transition states with the GlcNAc sugar in an envelope conformation, with conformational itineraries ^1,4^*B*/^1^*S*_3_ → [^4^*E*]^‡^ → ^4^*C*_1_ (cyclization step) and ^4^*C*_1_ → [^4^*E*]^‡^ → ^1,4^*B*/^1^*S*_3_ (ring-opening step). Our structures of the oxazolinium-ion intermediate and the TS of the rate-limiting step (cyclization) match very well the structures of enzyme complexes with known OGA inhibitors (Figure S14). In particular, PUGNAc and PUGNAc–imidazole have the closest similarity to TS1, while Thiamet-G mimics the intermediate. It is expected that the detailed mechanism will provide useful information for the rational design of other inhibitors and activity-based probes to control the activity of hOGA in a more efficient way.

## Supporting Information Available

Glycopeptide synthesis, enzyme production and crystallization, X-ray data collection and structure refinement statistics, computational details (cyclization and ring-opening steps, GlcNAc conformational FEL, and MC_A_/MC_B_ conformational change), additional tables and figures as described in the text (PDF)
