# Supplementary material for: Human O-GlcNAcase Uses a Preactivated Boat-skew Substrate Conformation for Catalysis. Evidence from X-ray Crystallography and QM/MM Metadynamics
Source: ACS Catal. 2023 Oct 10;13(20):13672–8. doi: 10.1021/acscatal.3c02378 (PMC10636738; doi:10.1021/acscatal.3c02378)
Supplement: Supplementary file 1 — cs3c02378_si_002.pdf [file cs3c02378_si_002.pdf]

Supporting information

Human *O*-GlcNAcase Uses a Preactivated Boat-like Substrate Conformation for Catalysis. Evidence from X-ray Crystallography and QM/MM Metadynamics

Martín Calvelo[1], Alexandra Males[2], Matthew G. Alteen[3], Lianne I. Willems[2], David J. Vocadlo[3], Gideon J. Davies[2], Carme Rovira[1,4,*]

[1]Departament de Química Inorgànica i Orgànica & IQTCUB, Universitat de Barcelona, Martí i Franquès 1, 08028 Barcelona, Spain. [2]York Structural Biology Laboratory. Department of Chemistry, The University of York, Heslington, York, YO10 5DD, United Kingdom. [3]Department of Chemistry & Department of Molecular Biology and Biochemistry, Simon Fraser University, Burnaby, BC, Canada. [4]Institució Catalana de Recerca i Estudis Avançats (ICREA), Passeig Lluís Companys, 23, 08020 Barcelona, Spain.

*Corresponding author e-mail: c.rovira@ub.edu

Contents Page

1. Synthesis of CKII-Cys-GlcNAc glycopeptides S2
2. Enzyme production, crystallization, structure solution and refinement S3
3. Modelling of the cyclization step S3
4. Modelling of the ring opening step S7
5. Simulation data S8
6. References S8
7. Supporting table S12
8. Supporting figures (S1-S14) S13

1. Synthesis of CKII-Cys-GlcNAc glycopeptides

The CKII-Cys-GlcNAc glycopeptides used in this study was generated chemoenzymatically using recombinantly-expressed O-GlcNAc Transferase (OGT). The unmodified peptide was obtained commercially (Biomatik Corporation, Cambridge, ON) and 1-2 mg oligopeptide at a final concentration of 1 mM was incubated with UDP-GlcNAc (5 mM), OGT (1 µM, prepared as previously described[1]) and 2U shrimp alkaline phosphatase (New England Biolabs, Whitby, ON) in PBS at pH 7.2 containing 12.5 mM $MgCl_2$. Reactions were incubated at 37 °C for 4 h or overnight. Prior to purification, the reactions were terminated by heating at 95 °C for 10 min and then centrifuged at 13,000 x g for 2 minutes. The supernatants were recovered and were purified on an Agilent 1200 series HPLC equipped with an Agilent XDB-C18 Eclipse reversed-phase column (9.4 × 250 mm, 5 µ particle size). Glycopeptides were eluted using a mobile phase consisting of H_2O and CH_3CN with 0.1% trifluoroacetic acid over a gradient of 10 to 50% CH_3CN as appropriate. Fractions containing the product were lyophilized to yield up to 1 mg glycopeptide as a white powder. The glycopeptide was purified to >95% purity and analyzed by High Performance Liquid Chromatography-Electrospray Ionization Mass Spectrometry (LC-MS) in positive ion mode, as described below (Figure S1). Expected mass 1842.7 g/mol; observed: m/z 1843.73 $[M+H]^+$, 922.71 $[M+2H]^{2+}$.

LC-MS was performed using a Dionex UltiMate® 3000 Ci Rapid Separation LC system equipped with an UltiMate® 3000 photodiode array detector probing at 250–400 nm, coupled to an HCT ultra ETD II (Bruker Daltonics) ion trap spectrometer, using Chromeleon® 6.80 SR12 software (ThermoScientific), esquireControl version 6.2, Build 62.24 software (Bruker Daltonics), and Bruker compass HyStar 3.2-SR2, HyStar version 3.2, Build 44 software (Bruker Daltonics). Mass spectrometry data analysis was performed using ESI Compass 1.3 DataAnalysis, version 4.4 software (Bruker Daltonics). Prior to analysis, the peptide was dissolved in LC-MS grade water at a concentration of 1.5 mM. A sample of this solution (5 µL) was chromatographically analysed using an Accucore C18 column (50 x 2.1 mm, particle size 2.6 µM, Thermo Fisher Scientific). For the mobile phase, a linear gradient of CH_3CN in water (both containing 0.1% formic acid (v/v)) at a flow rate of 0.3 mL min^{-1} at RT was used. The gradient was started at 5% CH_3CN and finished at 95% over 7 minutes, followed by a linear step of 95% CH_3CN for 3 minutes.

2. Enzyme production, crystallization, structure solution and refinement

Crystals of WT hOGA, protein produced by co-expression of the glycoside hydrolase domain (residues 11-396) and the stalk domain (535-715), were grown using the crystallization conditions described in reference [2]: 0.14-0.18 M triammonium citrate pH 7.5 and 16-20% PEG3350, 30 mg mL^{-1} hOGA was supplemented with 5 mM TCEP. Crystals were seeded using a x20 diluted stock and the wells were set up in a ratio of 1:0.2:0.8 P:S:R. The complex between hOGA and the CKII-Cys-GlcNAc glycopeptide was obtained by soaking the glycopeptide into the crystals at a final concentration of 5 mM. The glycopeptidewas soaked into hOGA crystals at a final concentration of 5 mM for 5 days. After testing the crystals for diffraction in house, data was collected at Diamond using the I04-1 beamline. The diffraction images were integrated using the Xia2 pipeline[3] then further processed using the CCP4 software suite.[4] Initially data reduction using Aimless [5-6] was conducted. The resulting structure was obtained after several cycles of refinement in REFMAC[7-12] and model building in COOT.[13] CCP4mg was used to render the figures).[14] Data collection and structure processing statistics are provided in Table S1.

3. Modelling of the cyclization step.

3.1. System preparation:

The initial coordinates of the hOGA were taken from the X-ray structure reported here, adding the missing loops with the software Modeller.[15] The truncated model described in Roth et al.,[2] which comprises amino acids 11-396 (N-terminal fragment) and 535-715 (C-terminal fragment) and preserves the catalytic activity, was used. Since the enzyme only shows catalytic activity in the dimeric form, the two protein subunits were considered. The coordinates of the Ser-GlcNAc substrate were also taken from the crystal structure reported here, substituting the sulphur atom of the Cys residue by an oxygen atom. The solvent-exposed chain of the glycopeptide, which was not solved in the X-ray structure, was reconstructed from the crystal structure of D175N hOGA in complex with p53-Ser-GlcNAc (PDB: 5UN8, chain G).[16] The software Chimera was used for the overlap of both structures.[17] The protonation states of aspartate, glutamate and histidine residues were assigned using the software PropKa3,[18-19] as well as visual analysis of the environment of each residue, considering the optimal pH for the hOGA activity (≈ 6.5).[20] The acid/base

residue (D175) was taken as protonated, whereas the assisting amino acid (D174) was taken as deprotonated.

3.2. Classical MD simulations:

The initial system was prepared using the LeaP code, included in the AmberTools21 package.[21] The hOGA with the glycopeptide unit was solvated in a cubic box containing 57201 water molecules and 20 sodium ions, in order to neutralize the protein charge. The force fields FF14SB,[22] GLYCAM_06j-1[23] and TIP3P[24] were used for the protein, GlcNAc and water solvent molecules, respectively. MD simulations were performed in several steps. We started with an energy minimization, using the steepest descent and conjugate gradients methods, in three steps. First, we relaxed the solvent molecules, holding the protein and substrate fixed. Secondly, the protein was relaxed but keeping the two catalytic residues (D174 and D175) and the substrate fixed. Finally, the entire system was relaxed. The system was subsequently heated to 300 K by increasing the temperature in intervals of 50K over 50 ps runs. During the first run, the enzyme and the substrate were kept fixed, whereas only the backbone atoms were kept fixes in the subsequent heating runs. Afterwards, the water density was converged at 300 K by running a simulation in the NPT ensemble for 500 ps, keeping the restrains in the protein backbone and the interaction between the substrate and the catalytic residues (in particular, the distance between the carboxylate group of D174 and the NH group of the GlcNAc and between the proton of the carboxylic group of D175 and the glycosidic oxygen we kept at values ≤ 3 Å). The system was subsequently equilibrated in the NVT ensemble during 50 ns, preserving only the distance restraints. Finally, the system was simulated in the NVT ensemble with no restraints for 100 ns. Three replicas assigning different initial velocities were performed, and one of them was extended up to 1 μs (Figure S3). Two replicas of classical MD were also carried out using CKII-GlcNAc-Cys instead of the natural substrate, following the same protocol. In this case, the parameters of the GAFF[25] force-field were used for modelling the sugar link to the sulphur atom. All simulations were carried out with the AMBER20 software,[21] whereas analyses (Figures S3-S14) were carried out using VMD[25] and cpptraj.[26]

3.3. QM/MM MD simulations:

Two snap-shots of the previous classical MD simulation, in which the acid/base residue interacts either with the glycosidic oxygen or with the assistant residue (D174), (MC_A

and MC_B, respectively) were selected. QM/MM MD simulations, combining DFT-based Born-Oppenheimer MD with force-field MD, were carried out starting from each of these two snap-shots of the classical MD trajectory, using the software CP2K v9.1[27] coupled to PLUMED v2.8.[28] The QM region was selected as to include the Ser-GlcNAc unit and the side chain of the two catalytic residues (D174 and D175). Part of the side of chain of an active site lysine (K98) was also included in the QM region, resulting in a total of 55 atoms (Figure S5, left panel). The QM subsystem was enclosed in a 15.56 x 15.25 x 14.11 $Å^3$ cell. The remaining atoms were treated at the molecular mechanics (MM) level, whereas the dangling bonds between the QM and the MM region were capped with hydrogen atoms. The QM region was described at DFT level using the PBE functional,[29] in consistency with previous works describing GH reaction mechanisms and carbohydrate conformations,[30-32] using the dual basis set of Gaussians and plane-waves (GPW) formalism. The Gaussian triple-ζ valence polarized (TZV2P) basis set was used to expand the wave function, converging the electron density employing an auxiliary plane-wave basis set with a density cut-off of 300 Ry, along with GTH pseudopotentials.[33] The structure was first optimized by simulated annealing, followed by an unbiased QM/MM MD simulation at 300 K in the NVT ensemble for 5 ps, using a time step of 0.5 fs.

3.4. QM/MM metadynamics simulations of the MC_A / MC_B conformational change:

QM/MM metadynamics simulations[34-37] were carried out using CP2K v9.1 coupled to PLUMED v2.8.[28] Two collective variables were employed (Figure S11A). The first CV accounts for the interaction between D175 and the glycosidic oxygen as well as the loss of the hydrogen bond between D174 and D175. The second CV describes the torsion of the COOH group of D175. The time evolution of the CVs, together with the reconstructed FES, are shown in Figures S11B and S11C. Gaussian-like biasing potentials of height 1 kcal/mol and 0.1 and 0.05 c. v. u. width for CV1 and CV2, respectively, were added every 100 MD steps (50 fs). For increasing accuracy, the Gaussian height was decreased to 0.1 kcal/mol in the region near the TS. The simulation was stopped after one recrossing over the TS (2426 Gaussians deposited, corresponding to 121.3 ps).

3.5. Calculation of the GlcNAc conformational free energy landscape

The conformational free energy landscape (FEL) of the GlcNAc in the active site of hOGA was computed employing collective variables that are derived from the Cremer-

Pople puckering coordinates.[38] In particular, the Cartesian projection coordinates (see definition in reference[39]) divided by the Cremer-Pople puckering amplitude (Q) were used: CV1 = qx/Q, CV2 = qy/Q and CV3 = qz/Q. Two QM/MM metadynamics simulations were performed, starting from MC_A and MC_B, respectively. In both cases, the QM region included the Ser-GlcNAc unit (32 atoms) enclosed in a 13.41 x 10.80 x 13.39 Å^3 cell. The same protocol than in previous metadynamics has been followed for the equilibration of the system, using a plane-waves cutoff of 290 Ry. Gaussian-shaped potentials of height 1.2 kcal/mol were added every 30 fs with a width of 0.035, 0.030, 0.020 c. v. u. for qx/Q, qy/Q and qz/Q, respectively. To increase accuracy, the height was decreased to 0.6 kcal/mol after 300 ps (10000 potentials added). The simulation was stopped after extending the simulation 50 ps more. Reweighting into the θ and ϕ Cremer–Pople puckering coordinates[28] was performed with PLUMED.[28]

3.6. QM/MM metadynamics simulations of the cyclization step:

One snap-shot from the previous simulation, corresponding to MC_A (i.e. with the acid/base residue D175 oriented towards the glycosidic oxygen and the sugar adopts a boat-type conformation, $^{1,4}B$), was selected as initial structure for QM/MM metadynamics simulations[34-37] of the cyclization step. One collective variable, composed by four distances, was used to build the bias and reconstruct the free energy profile. To properly discriminate between reactants and products, the distances corresponding to bonds that are formed in the reactants state (MC_A) were defined as positive, whereas those corresponding to bonds being formed in the products of the half-reaction (*i.e.* the oxazoline/oxazolinium-ion intermediate, INT) were defined as negative, leading to the following expression for the CV: $CV = (d_{(COO-H)_{D175}} - d_{COOH_{D175}\cdots O1}) + (d_{C1-O1} - d_{C1\cdots O_{\mathrm{NHAc}}})$. The first pair of distances ($d_{(OH)_{D175}} - d_{O1\cdots H_{D175}}$) accounts for the protonation of the leaving group, whereas the other pair ($d_{C1-O1} - d_{C1\cdots O_{\mathrm{NAc}}}$) describes the intramolecular nucleophilic attack. The time evolution of the CV is shown in Figure S7. Gaussian-like biasing potentials of height 1 kcal/mol and width 0.1 c. v. u. were added every 100 MD steps (50 fs). For a better accuracy, the Gaussian height was decreased (0.5 kcal/mol) upon crossing and recrossing over the TS. Following literature recommendations,[40] the simulation was stopped after the recrossing over the TS, resulting in the addition of a total number of 1500 Gaussian functions (75 ps). The structure of the

TS was further confirmed by committor analysis. Analysis of the trajectory was carried out with PLUMED and home-made python scripts.

4. Modelling of the ring opening step

4.1. System preparation and classical MD simulations:

One snap-shot from the previous QM/MM metadynamics simulation, in which the oxazolinium ion was formed, was chosen as starting point for the simulation of the second step of the reaction mechanism. The leaving peptide chain was removed, and the system was re-solvated and neutralized in a cubic box containing 50394 water molecules and 18 sodium ions. The force fields FF14SB and TIP3P were used for the protein and water solvent molecules, respectively, whereas the oxazolinium ion was modelled using the GAFF[25] force-field, using the Antechamber tool[26] and RESP charges calculated with Gaussian09[27] at HF/6-31G* level. A classical MD simulation, using the same protocol than in the previous section, was carried out to relax the system (Figure S8). During the MD, one water molecule was held in the catalytic centre for the first 50 ns by restraining the distances with the C1 of the oxazolinium ion and the carboxylate group of D174 at a value ≤ 3 Å. An unrestrained MD was then carried out for 50 ns more. We computed the probability distribution of the water molecules in the catalytic centre by counting the number of waters in a sphere of radius of 2 Å located in the centre of the C1 of the oxazolinium ion and the two oxygen atoms of the carboxylate of D174.

4.2. QM/MM MD simulations:

A frame of the previous unrestrained trajectory with a water molecule displaying distances to the substrate and D174 suitable for the catalysis was selected as the initial structure for the following QM/MM calculations. The QM region was composed by 53 atoms (Figure S5, right panel): the oxazolinium ion, the side chain of the two assisting and acid/base residues (D174 and D175) and the neighbouring Lys (K98), as well as the catalytic water molecule. The QM region was enclosed in a 16.19 x 13.42 x 15.01 $Å^3$ cell. The same protocol as in the first catalytic step was adopted, using a cut-off of 350 Ry for the convergence of the electron density. The final structure of the unbiased QM/MM MD calculation was used to initiate the metadynamics simulations.

4.3. QM/MM metadynamics simulations of the ring opening:

As in the first reaction step, one collective variable defined by four distances was used to build the bias potential and reconstruct the free energy profile: [$CV = (d_{O_w-H_w} - d_{COO^-_{D175}\cdots H_w}) + (d_{C1-O_{NAc}} - d_{C1\cdots O_w})$]. Two of the distances describe the deprotonation of the water by the D175 acid/base residue ($d_{O_w-H_w} - d_{COO^-_{D175}\cdots H_w}$), whereas the other two take into account the attack of the water oxygen to the C1 of the oxazolinium ion ($d_{O_w-H_w} - d_{O_{D175}\cdots H_w}$). Gaussian-like biasing potentials of height 1 kcal/mol and width 0.1 c. v. u. were added every 100 MD steps (50 fs), decreasing the Gaussian height to 0.5 kcal/mol during crossing and recrossing over the TS for improving accuracy. The simulation was stopped after one recrossing over the TS, (66.5 ps of simulation, 1330 potentials added). The structure of the TS was further confirmed by committor analysis. The time evolution of the CV is shown in Figure S9.

5. Simulation data

Coordinate files and other simulation data can be found in the Zenodo repository (https://zenodo.org) at DOI: 10.5281/zenodo.7828194.

6. References

1. Shen, D. L.; Gloster, T. M.; Yuzwa, S. A.; Vocadlo, D. J., Insights into O-linked N-acetylglucosamine ([0-9]O-GlcNAc) processing and dynamics through kinetic analysis of O-GlcNAc transferase and O-GlcNAcase activity on protein substrates. *J. Biol. Chem.* **2012,** *287*, 15395-408.

2. Roth, C.; Chan, S.; Offen, W. A.; Hemsworth, G. R.; Willems, L. I.; King, D. T.; Varghese, V.; Britton, R.; Vocadlo, D. J.; Davies, G. J., Structural and functional insight into human O-GlcNAcase. *Nat. Chem. Biol.* **2017,** *13*, 610-612.

3. Winter, G., xia2: an expert system for macromolecular crystallography data reduction. *J. Appl. Cryst.* **2010,** *43*, 186-190.

4. Winn, M. D.; Ballard, C. C.; Cowtan, K. D.; Dodson, E. J.; Emsley, P.; Evans, P. R.; Keegan, R. M.; Krissinel, E. B.; Leslie, A. G.; McCoy, A.; McNicholas, S. J.; Murshudov, G. N.; Pannu, N. S.; Potterton, E. A.; Powell, H. R.; Read, R. J.; Vagin, A.; Wilson, K. S., Overview of the CCP4 suite and current developments. *Acta Crystallogr. D Biol. Crystallogr.* **2011,** *67*, 235-42.

5. Evans, P. R.; Murshudov, G. N., How good are my data and what is the resolution? *Acta Crystallogr D Biol Crystallogr* **2013,** *69*, 1204-14.

6. Evans, P. R., An introduction to data reduction: space-group determination, scaling and intensity statistics. *Acta Crystallogr. D Biol. Crystallogr.* **2011,** *67*, 282-92.

7. Murshudov, G. N.; Skubak, P.; Lebedev, A. A.; Pannu, N. S.; Steiner, R. A.; Nicholls, R. A.; Winn, M. D.; Long, F.; Vagin, A. A., REFMAC5 for the refinement of macromolecular crystal structures. *Acta Crystallogr. D Biol. Crystallogr.* **2011,** *67*, 355-67.

8. Murshudov, G. N.; Vagin, A. A.; Dodson, E. J., Refinement of macromolecular structures by the maximum-likelihood method. *Acta Crystallogr. D Biol. Crystallogr.* **1997,** *53*, 240-55.

9. Nicholls, R. A.; Long, F.; Murshudov, G. N., Low-resolution refinement tools in REFMAC5. *Acta Crystallogr. D Biol. Crystallogr.* **2012,** *68*, 404-17.

10. Vagin, A. A.; Steiner, R. A.; Lebedev, A. A.; Potterton, L.; McNicholas, S.; Long, F.; Murshudov, G. N., REFMAC5 dictionary: organization of prior chemical knowledge and guidelines for its use. *Acta Crystallogr. D Biol. Crystallogr.* **2004,** *60*, 2184-95.

11. Winn, M. D.; Murshudov, G. N.; Papiz, M. Z., Macromolecular TLS refinement in REFMAC at moderate resolutions. *Methods Enzymol.* **2003,** *374*, 300-21.

12. Pannu, N. S.; Murshudov, G. N.; Dodson, E. J.; Read, R. J., Incorporation of prior phase information strengthens maximum-likelihood structure refinement. *Acta Crystallogr. D Biol. Crystallogr.* **1998,** *54*, 1285-94.

13. Emsley, P.; Cowtan, K., Coot: model-building tools for molecular graphics. *Acta Crystallogr. D Biol. Crystallogr.* **2004,** *60*, 2126-32.

14. McNicholas, S.; Potterton, E.; Wilson, K. S.; Noble, M. E., Presenting your structures: the CCP4mg molecular-graphics software. *Acta Crystallogr. D Biol. Crystallogr.* **2011,** *67*, 386-94.

15. Šali, A.; Blundell, T. L., Comparative Protein Modelling by Satisfaction of Spatial Restraints. *J. Mol. Biol.* **1993,** *234*, 779-815.

16. Li, B.; Li, H.; Lu, L.; Jiang, J., Structures of human O-GlcNAcase and its complexes reveal a new substrate recognition mode. *Nat. Struct. Mol. Biol.* **2017,** *24*, 362-369.

17. Pettersen, E. F.; Goddard, T. D.; Huang, C. C.; Couch, G. S.; Greenblatt, D. M.; Meng, E. C.; Ferrin, T. E., UCSF Chimera—A visualization system for exploratory research and analysis. *J. Comput. Chem.* **2004,** *25*, 1605-1612.

18. Olsson, M. H. M.; Søndergaard, C. R.; Rostkowski, M.; Jensen, J. H., PROPKA3: Consistent Treatment of Internal and Surface Residues in Empirical pKa Predictions. *J. Chem. Theor. Comput.* **2011,** *7*, 525-537.
19. Søndergaard, C. R.; Olsson, M. H. M.; Rostkowski, M.; Jensen, J. H., Improved Treatment of Ligands and Coupling Effects in Empirical Calculation and Rationalization of pKa Values. *J. Chem. Theor. Comput.* **2011,** *7*, 2284-2295.
20. Çetinbaş, N.; Macauley, M. S.; Stubbs, K. A.; Drapala, R.; Vocadlo, D. J., Identification of Asp174 and Asp175 as the Key Catalytic Residues of Human O-GlcNAcase by Functional Analysis of Site-Directed Mutants. *Biochemistry* **2006,** *45*, 3835-3844.
21. Case, D. A.; Aktulga, H. M.; Belfon, K.; Ben-Shalom, I. Y.; Berryman, J. T.; Brozell, S. R.; Cerutti, D. S.; III, T. E. C.; Cisneros, G. A.; Cruzeiro, V. W. D.; Darden, T. A.; Duke, R. E.; Giambasu, G.; Gilson, M. K.; Gohlke, H.; Goetz, A. W.; Harris, R.; Izadi, S.; Izmailov, S. A.; Kasavajhala, K.; Kaymak, M. C.; King, E.; Kovalenko, A.; Kurtzman, T.; Lee, T. S.; LeGrand, S.; Li, P.; Lin, C.; Liu, J.; Luchko, T.; Luo, R.; Machado, M.; Man, V.; Manathunga, M.; Merz, K. M.; Miao, Y.; Mikhailovskii, O.; Monard, G.; Nguyen, H.; O'Hearn, K. A.; Onufriev, A.; Pan, F.; Pantano, S.; Qi, R.; Rahnamoun, A.; Roe, D. R.; Roitberg, A.; Sagui, C.; Schott-Verdugo, S.; Shajan, A.; Shen, J.; Simmerling, C. L.; Skrynnikov, N. R.; Smith, J.; Swails, J.; Walker, R. C.; Wang, J.; Wang, J.; Wei, H.; Wolf, R. M.; Wu, X.; Xiong, Y.; Xue, Y.; York, D. M.; Zhao, S.; Kollman, P. A. *Amber 2020. University of California, San Francisco.*, 2021.
22. Maier, J. A.; Martinez, C.; Kasavajhala, K.; Wickstrom, L.; Hauser, K. E.; Simmerling, C., ff14SB: Improving the Accuracy of Protein Side Chain and Backbone Parameters from ff99SB. *J. Chem. Theor. Comput.* **2015,** *11*, 3696-713.
23. Kirschner, K. N.; Yongye, A. B.; Tschampel, S. M.; Gonzalez-Outeirino, J.; Daniels, C. R.; Foley, B. L.; Woods, R. J., GLYCAM06: a generalizable biomolecular force field. Carbohydrates. *J. Comput. Chem.* **2008,** *29*, 622-655.
24. Jorgensen, W. L.; Chandrasekhar, J.; Madura, J. D.; Impey, R. W.; Klein, M. L., Comparison of simple potential functions for simulating liquid water. *J. Chem. Phys.* **1983,** *79*, 926-935.
25. Humphrey, W.; Dalke, A.; Schulten, K., VMD: Visual molecular dynamics. *J. Mol. Graph.* **1996,** *14*, 33-38.

26. Roe, D. R.; Cheatham, T. E., 3rd, PTRAJ and CPPTRAJ: Software for Processing and Analysis of Molecular Dynamics Trajectory Data. *J. Chem. Theory Comput.* **2013,** *9*, 3084-95.

27. CP2K version 4.1, the CP2K developers group, 2016. CP2K is freely available from https://www.cp2k.org.

28. Tribello, G. A.; Bonomi, M.; Branduardi, D.; Camilloni, C.; Bussi, G., PLUMED 2: New feathers for an old bird. *Comp. Phys. Commun.* **2014,** *185*, 604-613.

29. Perdew, J. P.; Burke, K.; Ernzerhof, M., Generalized gradient approximation made simple. *Phys. Rev. Lett.* **1996,** *77*, 3865-3868.

30. Ardèvol, A.; Rovira, C., Reaction mechanisms in carbohydrate-active enzymes: glycoside hydrolases and glycosyltransferases. Insights from ab initio quantum mechanics/molecular mechanics dynamic simulations. *J. Am. Chem. Soc.* **2015,** *137*, 7528-7547.

31. Coines, J.; Raich, L.; Rovira, C., Modeling catalytic reaction mechanisms in glycoside hydrolases. *Curr. Opin. Chem. Biol.* **2019,** *53*, 183-191.

32. Morais, M. A. B.; Nin-Hill, A.; Rovira, C., Glycosidase mechanisms: Sugar conformations and reactivity in endo- and exo-acting enzymes. *Curr. Opin. Chem. Biol.* **2023,** *74*, 102282.

33. Goedecker, S.; Teter, M.; Hutter, J., Separable dual-space Gaussian pseudopotentials. *Phys. Rev. B* **1996,** *54*, 1703-1710.

34. Laio, A.; Parrinello, M., Escaping free-energy minima. *Proc. Natl. Acad. Sci. USA* **2002,** *99*, 12562-12566.

35. Iannuzzi, M.; Laio, A.; Parrinello, M., Efficient exploration of reactive potential energy surfaces using Car-Parrinello molecular dynamics. *Phys. Rev. Lett.* **2003,** *90*, 238302.

36. Ensing, B.; De Vivo, M.; Liu, Z. W.; Moore, P.; Klein, M. L., Metadynamics as a tool for exploring free energy landscapes of chemical reactions. *Acc. Chem. Res.* **2006,** *39*, 73-81.

37. Barducci, A.; Bonomi, M.; Parrinello, M., Metadynamics. *WIREs Comput. Mol. Sci.* **2011,** *1*, 826-843.

38. Cremer, D.; Pople, J. A., General definition of ring puckering coordinates *J. Am. Chem. Soc.* **1975,** *97*, 1354-1358.

39. Iglesias-Fernandez, J.; Raich, L.; Ardèvol, A.; Rovira, C., The complete conformational free-energy landscape of β-xylose reveals a two-fold catalytic itinerary for β-xylanases. *Chem. Sci.* **2015,** *6*, 1167-1177.
40. Ensing, B.; Laio, A.; Parrinello, M.; Klein, M. L., A recipe for the computation of the free energy barrier and the lowest free energy path of concerted reactions. *J Phys Chem B* **2005,** *109*, 6676-87.

7. Supporting table

Table S1. Data collection and structure processing statistics. Values in parentheses represent high resolution data.

**Data collection**	
PDB code 8P0L	hOGA_CKII-Cys-GlcNAc
**Data collection**	
Beamline	Diamond I04-1
Wavelength	0.9159
Space group	$P\ 4_3\ 2_1\ 2$
Cell dimensions:	
a, *b*, *c* (Å)	101.5, 101.5, 284.6
α, β, γ (°)	90.00
Resolution (Å)	284.62-2.50 (2.58-2.50)
R_{merge}	0.10 (0.87)
R_{pim}	0.03 (0.22)
CC(1/2)	1.00 (0.84)
$I / \sigma I$	14.4 (2.5)
Completeness (%)	100.0 (100.0)
Redundancy	15.7 (16.5)
**Refinement**	
Resolution (Å)	284.62-2.50 (2.58-2.50)
No. reflections	52629
R_{work} / R_{free}	0.22/0.27
No. atoms	
Protein	6960
Ligand	14
Water	26
B-factors (Å^2)	
Protein	74
Ligand	39
Water	54
R.m.s. deviations	
Bond lengths (Å)	0.009
Bond angles (°)	1.37
Ramachandran	
Favoured (%)	91.3
Outliers (%)	2.3

8. Supporting figures

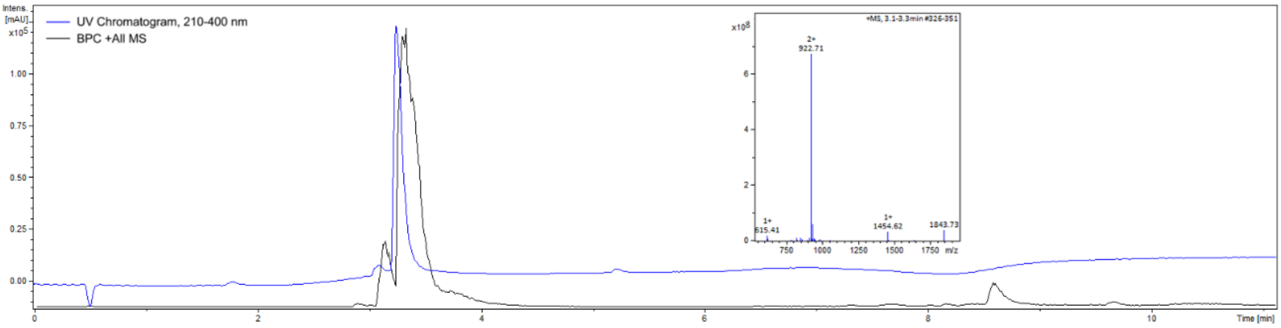

Figure S1. LC-MS analysis of CKII-Cys-GlcNAc glycopeptide (MW 1842.7). UV trace in blue, base peak chromatogram in black.

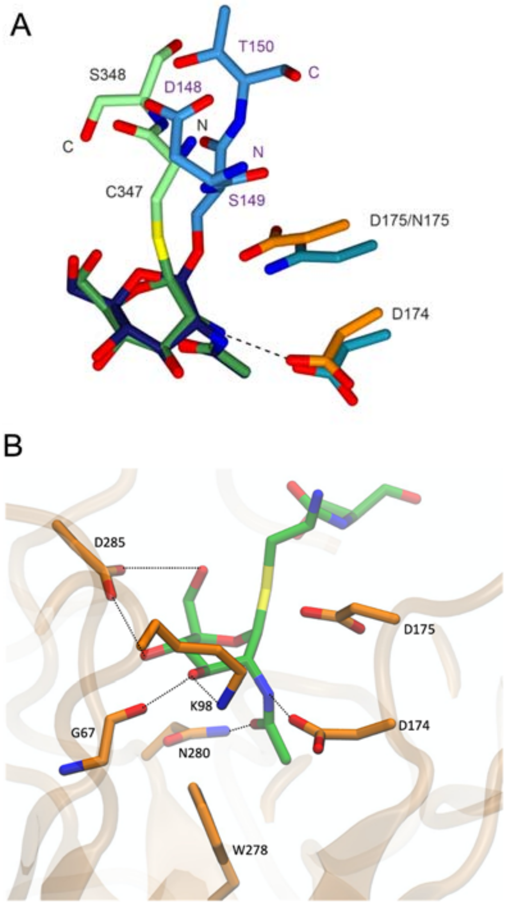

Figure S2. **A)** Superposition between the CKII-Cys-GlcNAc peptide, described in this work, and p53-GlcNAc peptide (PDB ID: 5UN8) bound to D175N hOGA. Both glycopeptides bind in a similar way and are shown in shades of green and blue, respectively. **B)** Stabilizing interactions between GlcNAc and hOGA observed in the X-ray structure.

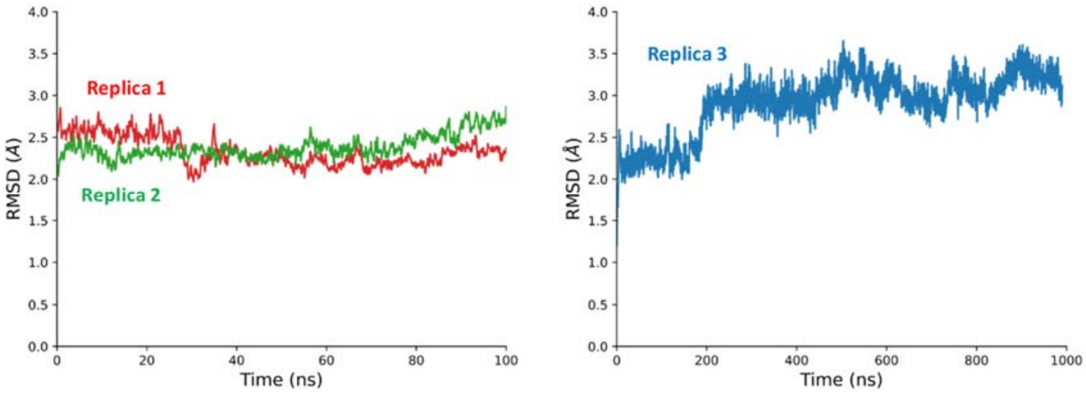

Fig S3. RMSD evolution during the classical MD simulations of the hOGA Michaelis complex.

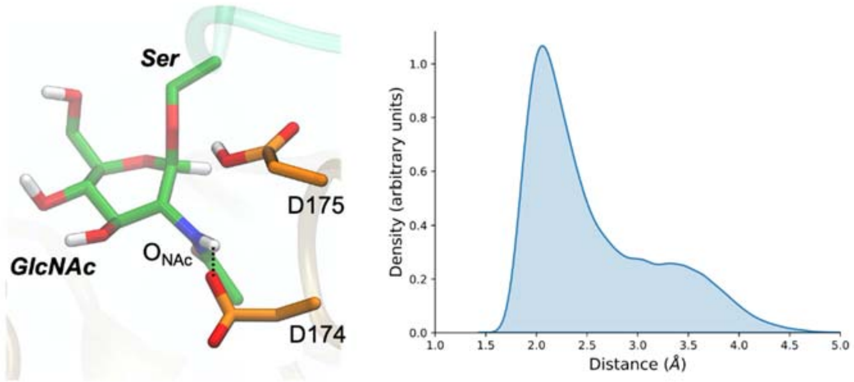

Fig S4. Probability distribution of the H-O distance corresponding to the hydrogen bond between D174 and the NH group of the acetamido substituent of GlcNAc during the classical MD simulations (the three replicas have been taken into account).

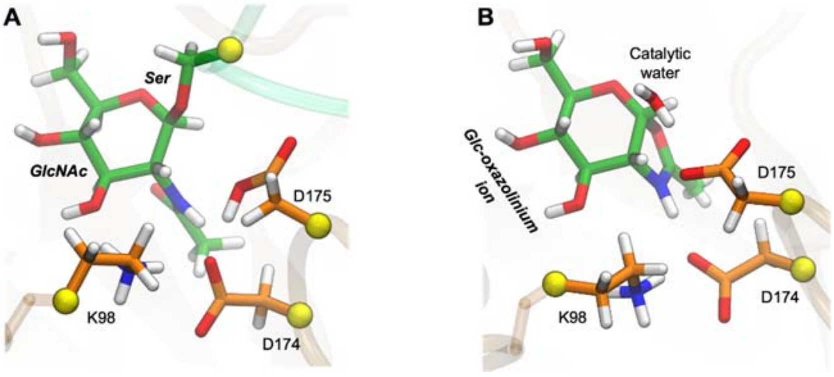

Fig S5. QM region (solid colour) used in the QM/MM simulations for the **A)** cyclization and **B)** ring opening step. Yellow spheres correspond to the capping hydrogen atoms.

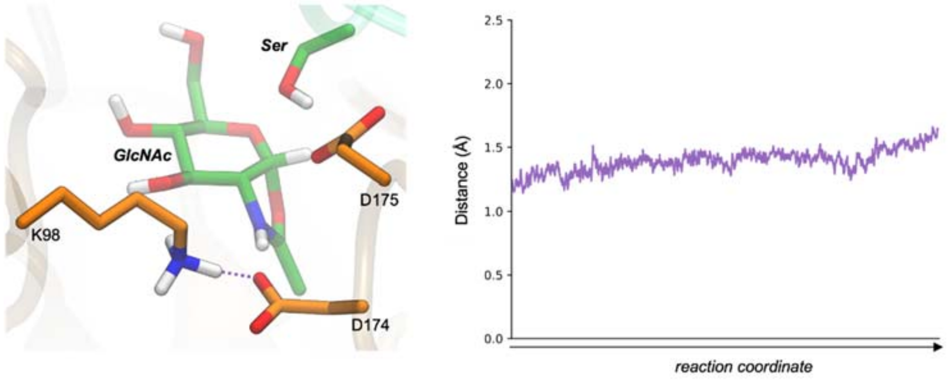

Fig S6. Interaction between the K98 and D174 along the reaction coordinate of the cyclization step, computed by QM/MM metadynamics (the left-hand picture represents the end-point of the reaction coordinate).

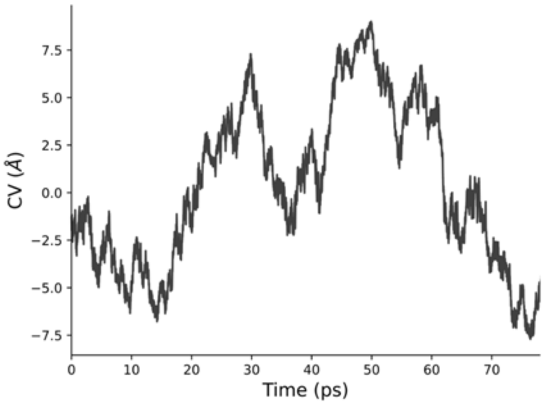

Fig S7. Time evolution of the CV employed in the QM/MM metadynamics of the cyclization step.

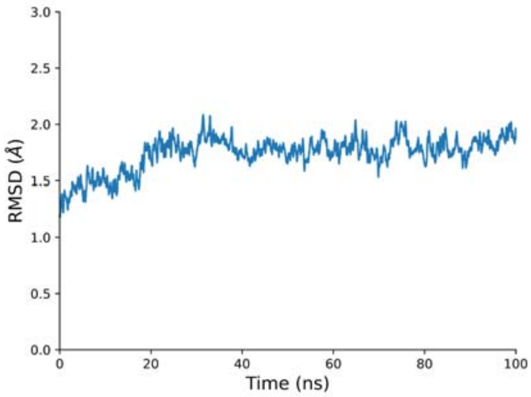

Fig S8. RMSD evolution during the classical MD simulation of the oxazolinium-ion intermediate (INT).

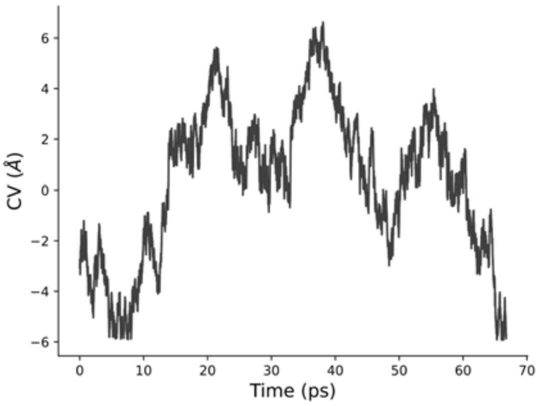

Fig S9. Time evolution of the CV employed in the QM/MM metadynamics for the ring opening step.

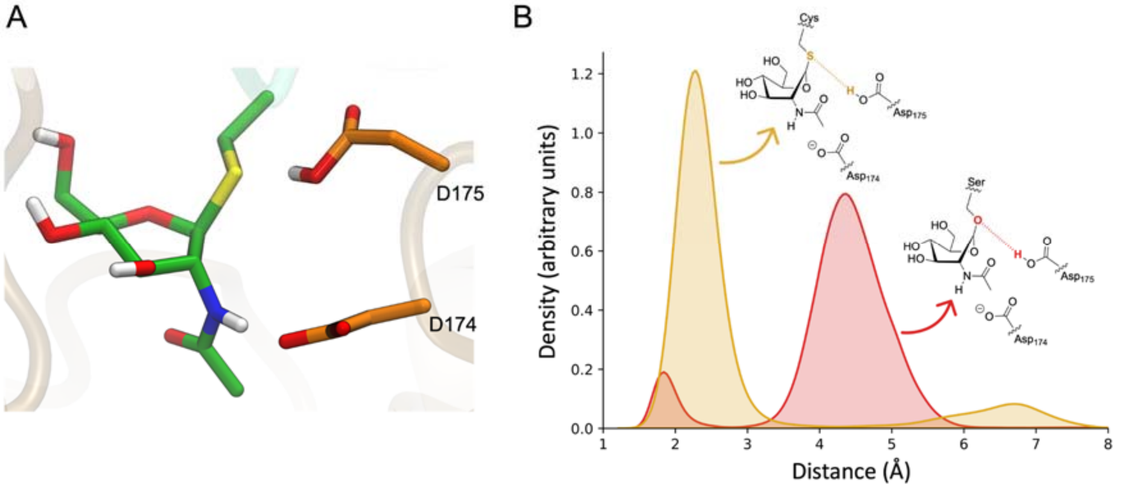

Fig S10. A) Representative structure of the classical MD simulation of the enzyme complex with CKII-GlcNAc-Cys. **B)** Density distribution of the distance between the glycosidic oxygen (simulation with the natural substrate) or the S atom (simulation with CKII-GlcNAc-Cys) and the closest H atom of the carboxylic group of D175.

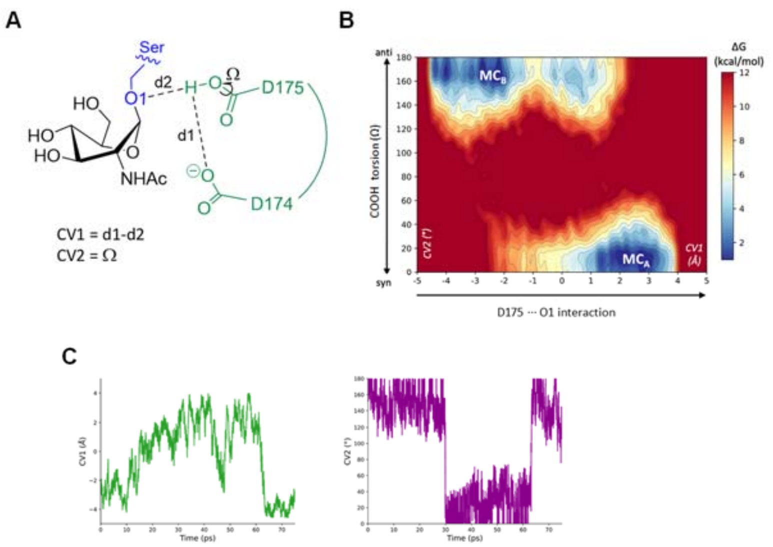

Fig S11. A) Collective variables used for the QM/MM metadynamics simulation of the interconversion between MCA and MCB. **B)** Reconstructed free energy landscape. **C)** Time evolution of the collective variables.

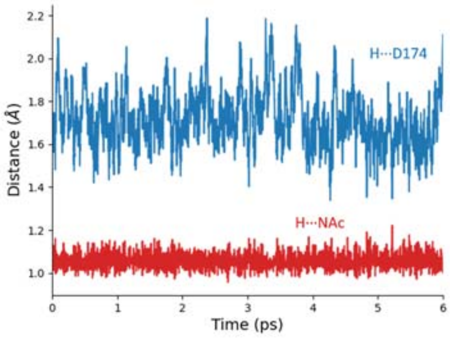

Fig S12. Time evolution of the H-NAc and H-D174 distance in an unbiased QM/MM simulation starting from INT.

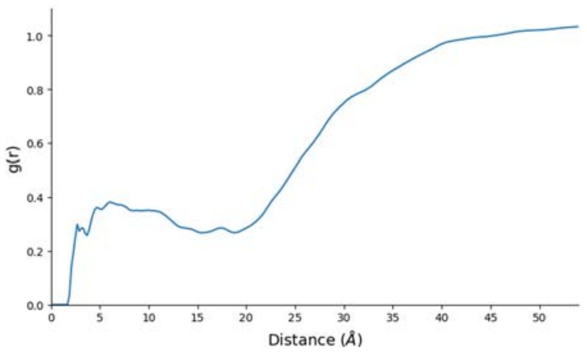

Fig S13. Radial distribution function of the water molecules around a reference point defined as the geometrical center between the C1 atom of GlcNAc and the carboxylate group of D175.

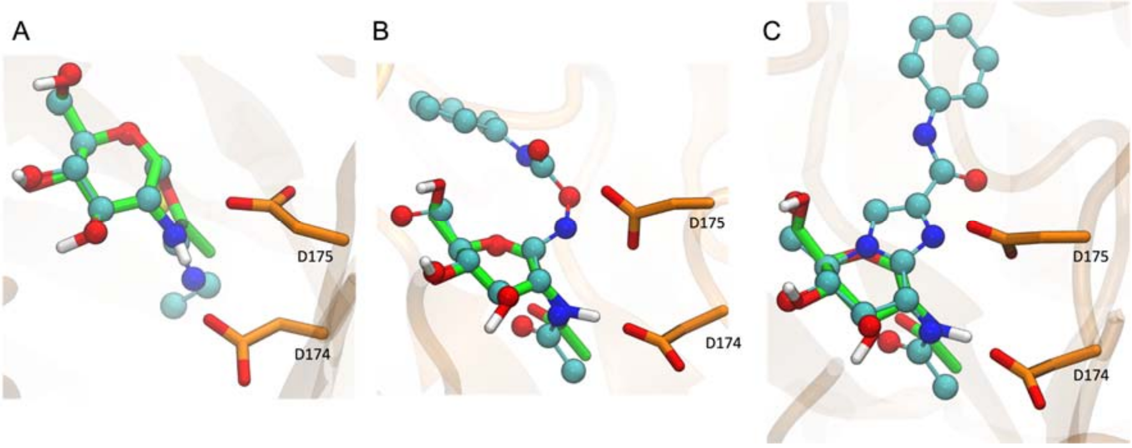

Fig S14. Overlapping of the structure of **A)** INT with hOGA in complex with Thiamet-G (PDB ID: 5M7S), **B)** TS1 with hOGA in complex with PUGNAc (PDB ID: 5UHO) and **C)** TS1 with hOGA in complex with PUGNAc-imidazole (PDB ID: 5M7T). The species from the natural reaction are shown in green, whereas the ones corresponding with the inhibitors are represented in cyan balls and sticks.
